# Internet-Based home care education for children with pulmonary hypertension: a public health perspective

**DOI:** 10.3389/fped.2026.1759723

**Published:** 2026-08-18

**Authors:** Yan Li, Juan Huang, Xuan Zhang, Yanhong Liu, Qingqing Song

**Affiliations:** Department of Cardiology, The Affiliated Children’s Hospital of Xiangya School of Medicine, Central South University (Hunan Children’s Hospital), Changsha, China

**Keywords:** digital health, family-centered care, health equity, home care education, pediatric pulmonary hypertension, public health intervention, telemedicine

## Abstract

**Background:**

Pediatric pulmonary hypertension (PH) requires complex, specialized home care. Digital health interventions offer scalable solutions to address healthcare disparities, improve educational access, and reduce health system burdens while empowering caregivers.

**Objective:**

This mini-review evaluates internet-based home care education for pediatric PH, focusing on public health implications, implementation strategies, and population-level outcomes.

**Methods:**

We conducted a comprehensive narrative review of recent literature on internet-based education platforms and telehealth solutions for pediatric pulmonary hypertension, evaluating them through public health frameworks of accessibility, equity, and scalability.

**Key findings:**

Internet-based programs significantly improve medication adherence, complex skill acquisition (e.g., central line care), early symptom recognition, and caregiver self-efficacy. Effective platforms leverage multimodal learning, adaptive technologies, and interactive features. From a public health perspective, these interventions reduce preventable emergency department visits, lower long-term healthcare costs, and enhance quality of life. However, major barriers persist, including the digital divide, variable digital health literacy, and a lack of standardized content validation.

**Conclusions:**

Internet-based home care education is vital for pediatric PH management. Future efforts must prioritize standardized, evidence-based curricula, health equity across socioeconomic strata, digital health reimbursement policies, and integration into existing public health infrastructure to maximize population-level benefits.

## Introduction

Pediatric pulmonary hypertension (PH) represents a heterogeneous group of disorders characterized by elevated pulmonary arterial pressure, affecting approximately 2–16 per million children worldwide (1). The complexity of PH management, coupled with its significant morbidity and mortality, poses substantial challenges for healthcare systems globally (2). Unlike many other chronic conditions, PH demands an exceptionally high level of caregiver technical competence; children require continuous monitoring, complex medication regimens often delivered via continuous intravenous or subcutaneous infusion, and immediate recognition of clinical deterioration, placing an immense cognitive and emotional burden on families and caregivers (3).

The traditional model of PH care, centered on specialized tertiary care facilities, often creates logistical barriers to optimal disease management, particularly for families residing in remote or underserved areas (4). These geographic and socioeconomic disparities in access to specialized PH care have been associated with poorer clinical outcomes and reduced quality of life (5). From a public health perspective, addressing these inequities while ensuring high-quality, continuous education for families represents a critical challenge in pediatric chronic disease management (6).

The digital transformation of healthcare, accelerated by the COVID-19 pandemic, has created unprecedented opportunities for reimagining pediatric PH care delivery (7). Internet-based educational platforms offer potential solutions to bridge gaps in care continuity, enhance family empowerment, and improve population-level outcomes (8). These digital interventions align with public health priorities by potentially reducing healthcare costs associated with avoidable complications, preventing hospital readmissions, and promoting health equity through improved democratization of specialized knowledge (9).

This review examines the current landscape of internet-based home care education for children with PH through a public health lens. We analyze existing digital educational interventions, evaluate their effectiveness and accessibility, identify implementation barriers, and propose strategies for optimizing population-level impact. By synthesizing current evidence and identifying gaps, this review aims to inform policy decisions and guide future development of digital health strategies for pediatric PH management.

## Methods and search strategy

A narrative literature search was conducted across PubMed, Scopus, and CINAHL for articles published between January 2015 and May 2025. Search terms included combinations of (“pediatric pulmonary hypertension” OR “children with PH”) AND (“internet-based” OR “digital health” OR “telemedicine” OR “home care education” OR “mobile health”). The time frame (2015–2025) was selected because 2015 represents a pivotal milestone marked by the publication of the landmark AHA/ATS Pediatric Pulmonary Hypertension guidelines, which coincided with the rapid technological acceleration of modern mHealth platforms and interactive remote care delivery.

### The public health burden of pediatric PH

#### Epidemiology and healthcare utilization

Pediatric PH imposes a significant burden on healthcare systems worldwide. Recent epidemiological data suggest an increasing prevalence, partly attributed to improved survival of premature infants and children with congenital heart disease (10). The annual healthcare costs for a child with PH can exceed $10,000, with frequent hospitalizations accounting for the majority of expenses (11). Emergency department visits for PH-related complications such as catheter-associated infections or pump malfunctions have increased over the past decade, highlighting the need for improved home competency and management strategies (12).

The complexity of PH management requires multidisciplinary care coordination, including pediatric cardiologists, pulmonologists, pharmacists, and specialized nurses (13). However, the distribution of PH expertise is highly concentrated in urban academic centers, creating significant access disparities. Studies indicate that children living in rural areas have higher rates of disease-related complications and reduced survival rates (14). This “zip code lottery” highlights a failure of traditional healthcare delivery models to support dispersed populations dealing with rare diseases.

#### Family and caregiver impact

The psychosocial burden on families caring for children with PH is substantial and often underrecognized from a public health perspective. Parents report high levels of anxiety, depression, and post-traumatic stress related to the fear of device failure or sudden cardiac events. The economic impact extends beyond direct medical costs, with many families experiencing job loss, reduced work hours, and financial instability (4).

Caregiver education and confidence in disease management directly correlate with child health outcomes (15). Families must master complex skills including sterile preparation of prostacyclins, oxygen therapy management, recognition of clinical deterioration, and emergency response protocols (16). Traditional education models, relying on brief hospital-based training sessions often delivered during stressful inpatient admissions, often prove insufficient for long-term retention and skill maintenance (17).

#### Health disparities and social determinants

Social determinants of health significantly influence pediatric PH outcomes. Children from racial and ethnic minority backgrounds experience higher rates of delayed diagnosis, reduced access to advanced therapies, and poorer long-term outcomes (18). Language barriers, health literacy limitations, and cultural factors further compound these disparities (19). Rural populations face unique challenges, including limited internet connectivity, reduced access to specialty care, and geographic isolation from support networks (20).

The intersection of chronic disease management and social vulnerability creates a compelling case for innovative, accessible educational interventions. Public health frameworks emphasize the importance of addressing these disparities through scalable, culturally appropriate interventions that reach vulnerable populations (21, 22).

### Evolution of digital health in pediatric chronic disease management

#### Historical context and technological advancement

The integration of digital health technologies in pediatric care has evolved rapidly over the past two decades. Early initiatives focused primarily on telemedicine consultations and basic web-based information resources. The proliferation of smartphones and high-speed internet has enabled more sophisticated, interactive educational platforms capable of delivering personalized content and real-time support (23). Crucially for PH, the shift from static text to video-based content has been vital for teaching dynamic skills, such as pump programming.

The COVID-19 pandemic served as a catalyst for digital health adoption, with pediatric telehealth visits increasing dramatically during initial lockdown periods (24, 25). This rapid transition demonstrated both the feasibility and acceptability of remote care delivery for chronic disease management. Families reported high satisfaction with virtual education sessions, citing convenience, reduced travel burden, and increased participation of multiple family members (26).

#### Evidence from other pediatric chronic conditions

Successful implementation of internet-based education in other pediatric chronic conditions provides valuable insights for PH management. In pediatric diabetes, digital education platforms have demonstrated significant improvements in glycemic control, self-management skills, and quality of life (27). Interactive mobile applications for asthma management have reduced emergency department visits and improved medication adherence rates (28).

Systematic reviews of digital health interventions in pediatric chronic disease consistently identify key success factors: user-friendly interfaces, age-appropriate content, gamification elements, and interoperable integration with clinical care teams (29). Programs incorporating behavioral change theories and personalized feedback mechanisms show superior outcomes compared to static information delivery (30).

#### Theoretical frameworks for digital health education

The application of health behavior theories to digital education design enhances effectiveness and engagement. The Social Cognitive Theory emphasizes the role of self-efficacy, observational learning, and reciprocal determinism in behavior change (31). Digital platforms can leverage these principles through video demonstrations, peer support forums, and progressive skill-building modules.

The Chronic Care Model, adapted for pediatric populations, provides a framework for integrating digital education within comprehensive care systems (32). This model emphasizes productive interactions between informed, activated families and prepared, proactive healthcare teams. Internet-based platforms serve as a bridge, facilitating continuous communication and support beyond traditional clinical encounters.

### Current landscape of internet-based PH education programs

#### Existing platforms and interventions

Several internet-based educational initiatives have emerged specifically for pediatric PH management. The PH Association's pediatric resources provide age-appropriate educational materials, including animated videos explaining disease pathophysiology and treatment options (33). These resources, available in multiple languages, address diverse literacy levels and cultural backgrounds.

Hospital-based programs have developed proprietary digital education platforms integrated with electronic health records (EHR). Preliminary data suggest improved medication adherence rates and reduced readmission rates among participating families (34). However, these systems are often siloed, limiting their utility for patients who receive care across multiple institutions.

Commercial telehealth platforms have expanded to include specialized PH education modules (35). Platforms like MyPHteam offer social networking features alongside educational content, creating virtual support communities for affected families. These platforms facilitate peer-to-peer learning and emotional support, addressing the isolation often experienced by families managing rare diseases. However, the rise of unmoderated social platforms also introduces public health risks related to the spread of anecdotal, non-evidence-based medical advice.

#### Content development and standardization

The development of evidence-based educational content for pediatric PH remains challenging due to disease heterogeneity and rapidly evolving treatment paradigms (36). Current programs vary significantly in scope, depth, and quality, highlighting the need for standardized curricula. The World Symposium on PH has proposed core educational competencies for family caregivers, including medication management, symptom recognition, emergency response, and psychosocial support (37).

Successful programs employ multidisciplinary teams in content development, including pediatric PH specialists, nurses, pharmacists, child life specialists, and family advisors (38). The incorporation of family perspectives ensures relevance and usability of educational materials. Visual learning aids, including infographics, decision trees, and interactive anatomical models, enhance comprehension of complex medical concepts (39).

#### Delivery methods and engagement strategies

Internet-based PH education programs utilize diverse delivery methods to accommodate varying learning preferences and technological capabilities. Synchronous approaches include live webinars, virtual support groups, and one-on-one video consultations with healthcare providers. These real-time interactions facilitate immediate question resolution and personalized guidance (40).

Asynchronous methods offer flexibility for families managing complex schedules and time zone differences. Self-paced learning modules, recorded video tutorials, and downloadable resources allow families to access education at convenient times. This flexibility is particularly crucial for caregivers of children with chronic conditions, who often experience high levels of time-burden and stress (41). Microlearning strategies—delivering content in brief, focused segments—are increasingly recommended in medical education to improve retention and reduce cognitive overload for non-medical caregivers (42).

While still emerging in the specific field of PH, gamification elements and Virtual Reality (VR) are gaining traction in broader pediatric cardiology. VR simulations allow families to visualize complex heart defects and practice care scenarios in safe, controlled environments, a method that has proven effective in congenital heart disease education and is applicable to high-risk procedures in PH management (43).

#### Caregiver empowerment, disease stratification, and triage criteria

**Figure 1:**
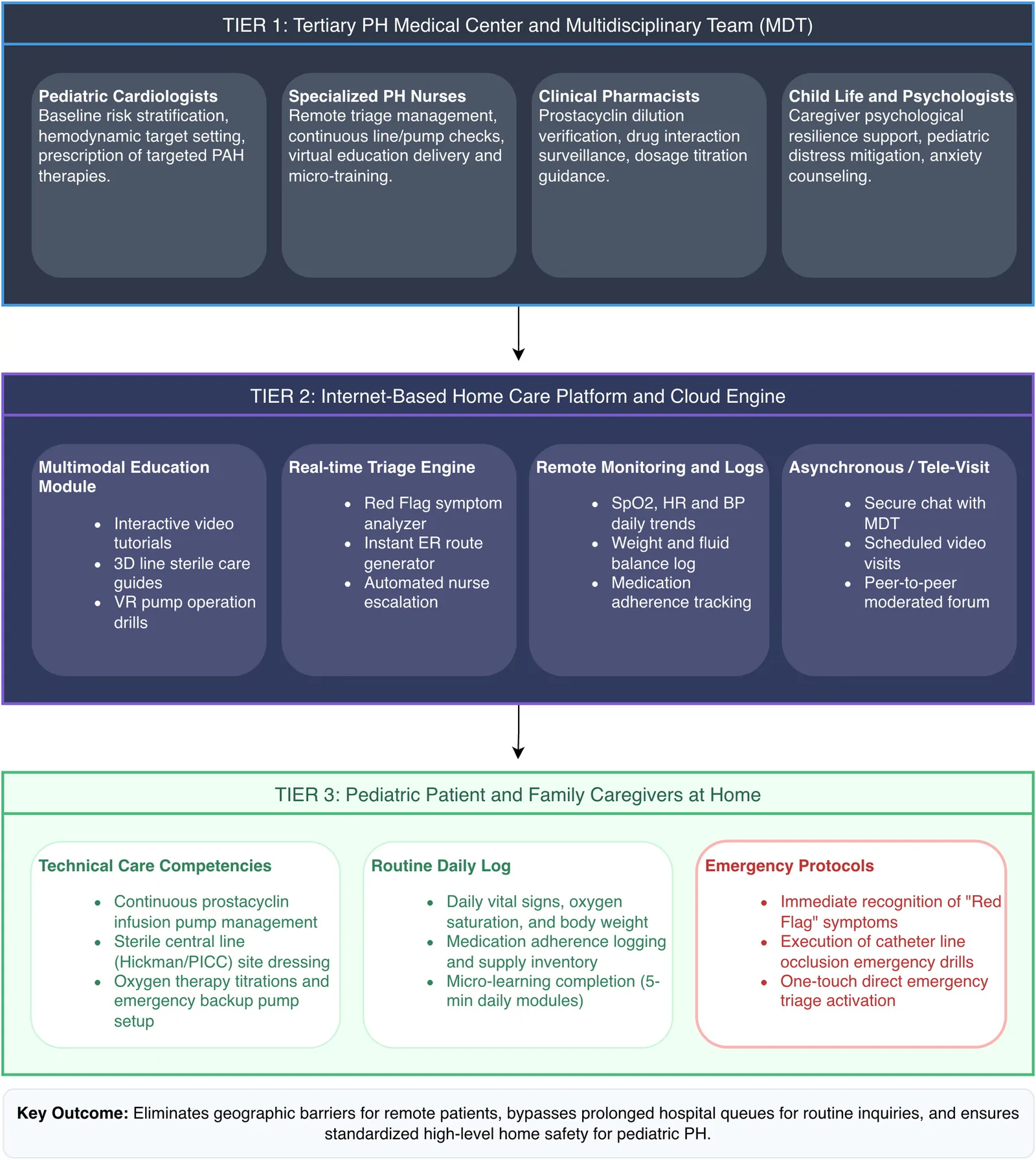
Implementation framework of internet-based home care education for pediatric pulmonary hypertension. A structured three-tier model illustrating the hierarchical flow of remote pediatric PH management and caregiver empowerment. Tier 1 (top) represents the specialized tertiary medical center housing a multidisciplinary team responsible for clinical diagnosis, complex prescriptions, and specialized education. Tier 2 (middle) depicts the cloud-based digital health infrastructure providing interactive modules, real-time triage algorithms, continuous monitoring logs, and tele-visit capabilities. Tier 3 (bottom) details home-based caregiver core competencies, daily logging protocols, and emergency response mechanisms. This integrated framework eliminates geographic barriers and reduces preventable hospital transfers. MDT, multidisciplinary team; PAH, pulmonary arterial hypertension; EHR, electronic health record; ER, emergency room; VR, virtual reality; 3D, three-dimensional; SpO2, peripheral oxygen saturation; HR, heart rate; BP, blood pressure; PICC, peripherally inserted central catheter.

Caregivers play an indispensable operational and clinical role in the continuous management of pediatric PH. Within the home environment, family members act as primary care managers responsible for executing technically complex daily protocols. These duties range from the sterile preparation and precise administration of continuous intravenous or subcutaneous prostacyclins to pump maintenance, central line care, and the systematic tracking of daily vital signs. Given the high cognitive and emotional burden associated with these procedures, empowering caregivers through structured, accessible internet-based education is critical to improving caregiver self-efficacy, reducing administration errors, and ensuring long-term patient stability. To support these daily responsibilities, an integrated internet-based home care implementation framework provides structured clinical support, educational modules, and real-time guidance (Figure 1). Global experiences further demonstrate that structured digital empowerment can significantly improve caregiver competency while reducing overall healthcare utilization across diverse socioeconomic settings (Table 1).

**Table 1:** Cross-country comparison of internet-based home care and telehealth implementations for pediatric pulmonary hypertension and chronic pediatric conditions.

Aspect/Region	United States (USA)	European Union (EU)	China	Low-and Middle-Income Countries (LMICs)
Primary Digital Interventions & Supported Treatments	Comprehensive EHR-integrated telehealth platforms, interactive apps (e.g., MyPHteam), continuous infusion pump remote tracking, and virtual VR/animation education.	National registry-backed remote monitoring systems, structured asynchronous caregiver training modules, and virtual multidisciplinary team consultations.	Hospital-centric mobile health (mHealth) applications, dedicated WeChat-based home care networks, and remote video triage platforms.	Basic SMS/WhatsApp-based care instructions, simplified video tutorials, and mobile hotline support networks.
Caregiver & Patient Satisfaction	High satisfaction due to reduced travel burdens and increased convenience; occasional friction stems from digital health literacy gaps.	High acceptance and measurable quality-of-life improvements, reinforced by strong family trust in centralized public healthcare frameworks.	High satisfaction among rural families by effectively bypassing long tertiary hospital queues and cutting travel expenses.	Moderate-to-high caregiver empowerment, though overall satisfaction is constrained by hardware and connectivity limitations.
Healthcare System & Financial Impact	Significant reduction in avoidable ED visits and readmissions; indirect cost savings (e.g., travel, missed work). Reimbursement varies by state/insurer.	Public health cost savings through reduced emergency bed occupancy. Reimbursed under standardized national digital health codes in select countries.	Substantial decrease in non-urgent outpatient clinic visits and reduced out-of-pocket transportation costs for rural families.	Low-cost setup yielding high relative economic benefits by preventing severe, costly complications in resource-limited settings.
Geographic Reach & Queue Mitigation	Mitigates “medical deserts” and long-distance travel burdens for geographically isolated rural populations.	Streamlines subspecialty referrals and facilitates cross-regional healthcare network integration.	Drastically cuts physical waiting queue times at top-tier urban medical centers by shifting routine education to the home.	Bridges extreme geographical isolation and partially offsets the critical shortage of pediatric PH specialists.
Key Challenges & Limitations	High cost of software development, strict data privacy regulations (HIPAA), and persistent digital divide barriers.	Cross-border regulatory variations, strict GDPR data privacy constraints, and multi-language translation needs.	Digital health literacy disparities across regions, with a heavy communication burden on tertiary care nursing staff.	Severe broadband and smart device limitations, alongside a lack of localized and standardized digital curricula.

A comprehensive comparative analysis across four representative socioeconomic regions (USA, EU, China, and LMICs), evaluating primary digital health modalities, caregiver satisfaction, healthcare system financial impacts, geographic reach, and key implementation challenges.

EHR, electronic health record; VR, virtual reality; mHealth, mobile health; ED, emergency department; GDPR, General Data Protection Regulation; HIPAA, Health Insurance Portability and Accountability Act; LMICs, low- and middle-income countries; PH, pulmonary hypertension.

To deliver targeted digital health education effectively, patients must be stratified according to disease severity and clinical risk profiles. Pediatric PH management utilizes risk stratification frameworks—categorizing patients into low, intermediate, or high-risk tiers based on WHO functional class, clinical evidence of right heart failure, and serial hemodynamic parameters. For low-risk patients receiving stable oral or inhaled therapies, internet-based education focuses primarily on asynchronous learning modules, digital medication adherence tracking, and routine virtual follow-ups. Conversely, intermediate-to-high-risk patients—particularly those dependent on continuous parenteral prostacyclin infusions or multi-drug combination regimens—require a more intensive digital health protocol. This includes continuous real-time remote monitoring, daily visual checks of infusion sites, interactive video consultations, and mandatory periodic caregiver simulation drills to preserve technical competence.

**Figure 2:**
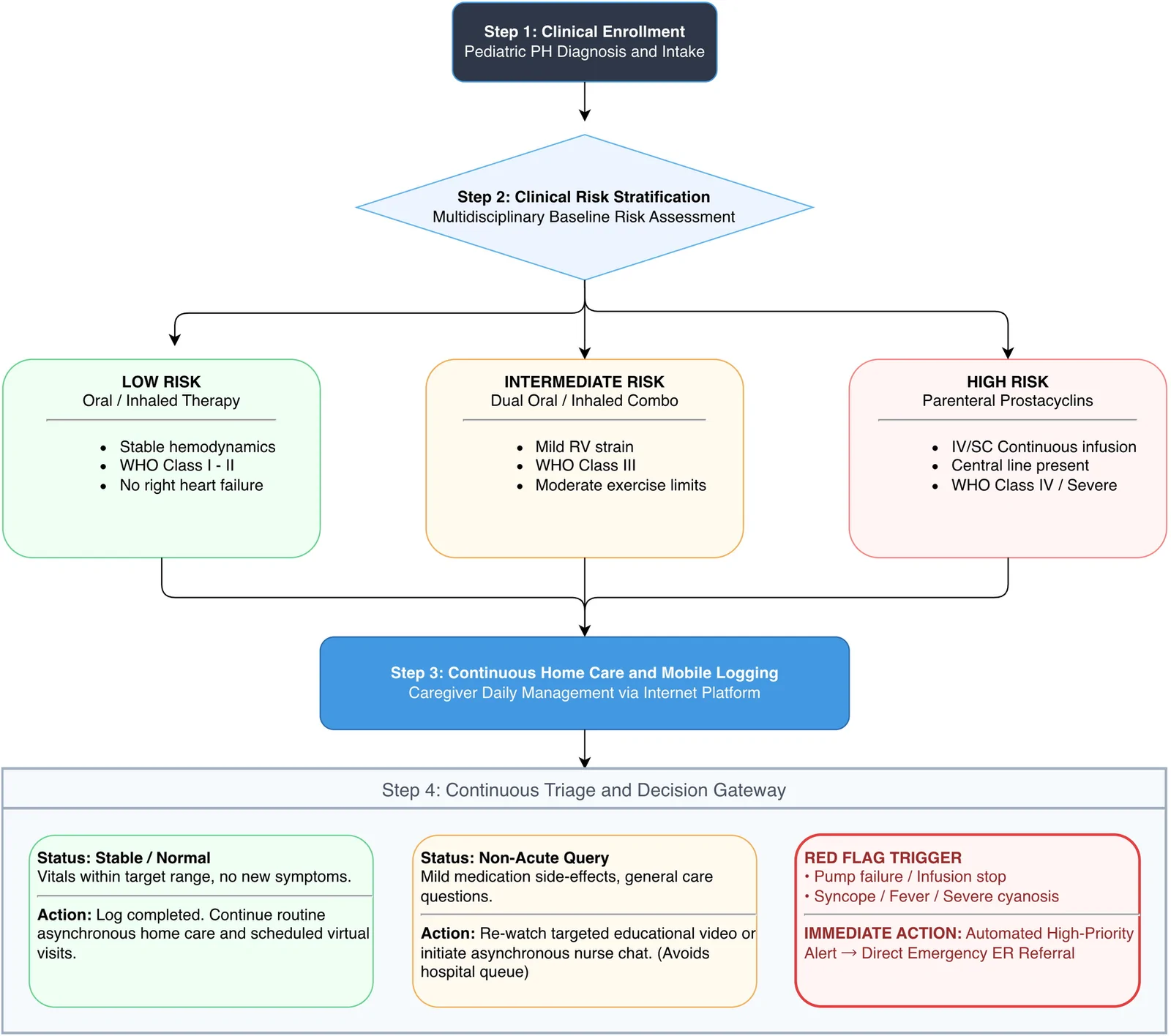
Proposed system flowchart for patient intake, disease severity stratification, and digital triage. A clinical decision-support pathway guiding patient management from initial enrollment through risk stratification to automated triage. Upon enrollment (Step 1), pediatric patients undergo multidisciplinary risk assessment (Step 2) categorizing them into Low Risk (oral/inhaled therapy), Intermediate Risk (dual therapy), or High Risk (parenteral prostacyclins). Following daily home logging via the internet platform (Step 3), the decision gateway (Step 4) automatically routes non-acute queries to educational refreshers/asynchronous nurse chat, while triggering immediate high-priority alerts for “red flag” clinical events (e.g., pump failure, syncope, severe cyanosis). WHO, world health organization; WHO Class, world health organization functional class; RV, right ventricle/right ventricular; IV, intravenous; SC, subcutaneous; ER, emergency room.

A fundamental function of the proposed digital platform is establishing unambiguous clinical triage criteria to assist caregivers in identifying when home management is no longer sufficient and immediate hospital care is required (Figure 2). The platform incorporates decision-support tools that prompt immediate emergency department referral upon the occurrence of specific “red flag” clinical triggers. These triggers are categorized into device-related compromises, cardiovascular deterioration, and severe respiratory or systemic events. Specifically, central venous catheter displacement, local line infections, infusion pump malfunctions, or any uncoordinated interruption of continuous prostacyclin delivery mandate immediate emergency evaluation. Furthermore, cardiovascular red flags such as recurrent syncope, unexplained lethargy, sudden severe cyanosis, or rapidly progressing peripheral edema necessitate urgent hospital admission. Acute dyspnea at rest, oxygen saturation dropping below personalized baseline targets, or high-grade fevers also trigger mandatory hospital referral protocols.

This structured digital triage and educational framework is particularly transformative for pediatric patients living in remote regions far from tertiary PH specialty centers. For these families, traditional care models present severe financial, geographical, and logistical barriers, often requiring long-distance travel only to encounter extended waiting queues at crowded subspecialty clinics. The proposed internet-based system bridges this gap by enabling remote clinical evaluation, asynchronous micro-learning, and virtual nurse-led triage. By addressing non-emergent clinical inquiries and routine educational reinforcement remotely, the platform minimizes unnecessary clinic visits, drastically reduces physical waiting queue times, and optimizes resource allocation within tertiary healthcare systems.

### Implementation challenges and barriers

#### Digital divide and access inequities

Despite the potential of internet-based education, significant barriers to equitable access persist. The digital divide disproportionately affects rural, low-income, and minority populations—groups that often experience a higher burden of cardiovascular disease complications (44). Families with children with chronic conditions may lack reliable broadband internet access, limiting their ability to participate in data-heavy digital health programs or video consultations (45).

Device availability presents another challenge. While smartphone ownership is widespread, complex educational platforms or remote monitoring dashboards often require larger screens or specific software incompatible with older devices. Research in digital health equity emphasizes that programs must consider these technological constraints in platform design to avoid excluding the most vulnerable populations (46).

Digital health literacy varies significantly across populations. Even with internet access, families may lack the skills to navigate complex educational platforms or evaluate the credibility of online health information. This is compounded by language barriers; a significant portion of digital health resources in pediatric cardiology are available only in English, limiting their utility for non-English speaking families who are already at risk for poorer health outcomes (47).

#### Privacy, security, and regulatory considerations

The collection of pediatric health information through digital platforms raises critical privacy concerns. Compliance with regulations such as HIPAA in the United States and GDPR in Europe is mandatory, yet families often express hesitation regarding data sharing, particularly concerning sensitive prognostic information (48). Furthermore, the reimbursement landscape for digital education remains fragmented. The lack of standardized billing codes for “patient education” compared to “clinical telemedicine visits” can discourage healthcare systems from investing in comprehensive educational platforms (49).

#### Sustainability and scalability

The long-term sustainability of internet-based PH education programs is often precarious. Many initiatives rely on short-term grant funding, creating vulnerability when funding cycles end. Without established reimbursement mechanisms for asynchronous digital education, maintaining these platforms becomes a financial burden for resource-constrained hospital systems (50).

Scalability is further hindered by the need for continuous content updates. As PH treatment guidelines evolve—such as the introduction of new vasodilators or changes in risk stratification—educational materials require regular revision to maintain clinical accuracy. The specialized nature of pediatric PH limits the potential user base compared to conditions like asthma or diabetes, affecting the economic feasibility of commercial platform development (5). Consequently, partnerships with patient advocacy groups and non-profit organizations are often essential to bridge the funding gap.

### Effectiveness and outcomes assessment

#### Clinical outcomes and healthcare utilization

Emerging evidence supports the positive impact of remote management and education on clinical outcomes. In the context of pediatric PH, studies on home monitoring programs—which include an educational component—have demonstrated that regular digital tracking of physiological parameters can facilitate early detection of clinical deterioration (51). While direct data on “education-only” apps is limited, broader pediatric cardiology studies suggest that enhanced caregiver competence correlates with reduced unplanned hospitalizations (52).

#### Quality of life and knowledge acquisition

Quality of life (QoL) metrics show promising improvements following digital interventions. Parents of children with severe chronic illnesses report increased confidence in disease management and reduced feelings of isolation when connected to online support communities (53). Furthermore, interactive multimedia platforms have been shown to be superior to static text-based resources in enhancing knowledge retention regarding medication mechanisms and emergency protocols (54).

However, knowledge gaps persist. Complex topics such as the pathophysiology of pulmonary vascular resistance and specific drug-drug interactions remain challenging. Tailored education that adapts to the parent’s baseline health literacy via artificial intelligence algorithms has been identified as a necessary evolution for future platforms (55).

### Public health implications and policy considerations

#### Health equity and access to care

Internet-based education holds significant potential for addressing health disparities in pediatric PH. By eliminating geographic barriers, digital platforms can democratize access to specialized knowledge previously concentrated in tertiary care centers. This is particularly vital for families living in “medical deserts” where access to a PH specialist is physically distant (56).

However, to prevent the “digital inverse care law”—where those most in need have the least access—policies must support universal broadband and device lending programs. Public health initiatives should prioritize culturally adapted content that reflects the diversity of the patient population (57).

#### Healthcare system integration and efficiency

From a health system perspective, integrating education into the Electronic Health Record (EHR) offers opportunities for efficiency. EHR-integrated education allows providers to “prescribe” specific learning modules based on the child’s current clinical status and monitor family engagement. Economic analyses in chronic disease management suggest that the initial investment in these platforms can be offset by savings from reduced emergency department utilization and shortened lengths of stay during readmissions (58).

## Conclusions

Internet-based home care education represents a transformative approach to managing pediatric PH from a public health perspective. The evidence presented in this review suggests significant potential for improving caregiver confidence, facilitating early symptom recognition, and bridging geographic gaps in care. Digital platforms offer unique advantages in delivering continuous, personalized support that extends beyond the clinical encounter.

However, significant challenges regarding the digital divide, health literacy, and sustainable funding persist. Success depends on thoughtful integration with existing healthcare systems and a commitment to equity. Future directions must prioritize the development of standardized, evidence-based curricula and the use of emerging technologies like AI to curate and personalize the learning experience. Furthermore, stakeholders must address the validation of content to combat digital misinformation. Ultimately, investing in digital education aligns with the broader public health goal of empowering families with the knowledge and skills necessary for optimal disease management.
